# Human cytomegalovirus plasmid-based amplicon vector system for gene therapy

**DOI:** 10.1186/1479-0556-3-1

**Published:** 2005-01-26

**Authors:** Kutubuddin Mahmood, Mark N Prichard, Gregory M Duke, George W Kemble, Richard R Spaete

**Affiliations:** 1 MedImmune Vaccines Inc., 297 North Bernardo Avenue, Mountain View, CA 94043 USA

## Abstract

We have constructed and evaluated the utility of a helper-dependent virus vector system that is derived from Human Cytomegalovirus (HCMV). This vector is based on the herpes simplex virus (HSV) amplicon system and contains the HCMV orthologs of the two *cis*-acting functions required for replication and packaging of HSV genomes, the complex HCMV viral DNA replication origin (oriLyt), and the cleavage packaging signal (the *a *sequence). The HCMV amplicon vector replicated independently and was packaged into infectious virions in the presence of helper virus. This vector is capable of delivering and expressing foreign genes in infected cells including progenitor cells such as human CD34+ cells. Packaged defective viral genomes were passaged serially in fibroblasts and could be detected at passage 3; however, the copy number appeared to diminish upon serial passage. The HCMV amplicon offers an alternative vector strategy useful for gene(s) delivery to cells of the hematopoietic lineage.

## Background

HCMV is a member of the betaherpesvirus family [42,48]. Other members of this family include human herpesvirus 6 (HHV-6), and human herpesvirus 7 (HHV-7), and all are widely distributed in human populations. During productive replication, the 230 kilobase pair (kbp) viral genome replicates by a rolling circle mechanism, which generates long head-to-tail concatemers that are cleaved to unit length and packaged in capsids. The state of the HCMV genome during latency remains unidentified and is likely to be circular and extrachromosomal [6]. The HCMV genome has been detected in cells within the hematopoietic lineage as early as CD34+ progenitors and up through differentiated macrophages [23,29,38,54].

Defective HSV viruses created by high multiplicity serial passage of virus stocks have been described on numerous occasions and have been characterized in detail at the molecular level [13,18,31,43,52,67]. Naturally occurring defective HSV viruses and laboratory derived HSV amplicon vectors are composed of head-to-tail tandem reiterations of subgenomic regions containing a functional origin of DNA replication (Ori_S_or Ori_L_) and a DNA cleavage/packaging signal [3,4,30,57,60-62]. These two *cis*-acting functions can be relatively small ranging from *ca*. 90–150 base pairs (bp) for the ori and *ca*. 250–300 bp for the *a *sequence. The functional HCMV oriLyt is much more complex than either of the HSV oris; the HCMV oriLyt consists of multiple direct and inverted repeats and extends over at least 1500 bp [1,2,24,37]. HCMV is unique among the herpesviruses in not having an origin binding protein homolog that is required for DNA replication [45]. The HCMV *a *sequence varies in size from *ca*. 550 bp to 762 bp, however, the conserved pac-1 and pac-2 *cis*-elements which determine the sites for cleavage of replicated viral DNA are present [15,28,58,64,65].

In contrast to HSV, HCMV does not efficiently produce defective virus genomes, this difference may be related to the distinct biology of the two viruses [45]. However two reports described the identification of what may potentially be HCMV defective viruses created by serial high multiplicity passage [47,59]. These reports characterized HCMV defectives as very large subgenomic DNA molecules, in some cases extending over two thirds of the genome. In addition to these replication defective HCMV viruses, a recent report by Borst *et al*. 2003 [7], described the construction of an HCMV amplicon. In this report we further utilized the HCMV amplicon for gene delivery to human CD34+ cells.

HCMV infects cells of the hematopoietic lineage [34,38,39,55,68]. Viral genomes can be found in CD34+ cells from seropositive individuals and granulocyte-macrophage progenitors and differentiated macrophages can be infected experimentally [56]. We were interested in determining whether the tropism of HCMV can be exploited to construct defective HCMV virus vectors (amplicons) targeted to hematopoietic stem cells. The general feasibility of such an approach for other cell types has been shown using other herpesviruses, e.g. HSV, EBV, and HHV-7 [20,25,26,30,35,49,70,71].

## Methods

### Cells and virus

HCMV Toledo (passage 8, from Dr. S. Plotkin, Aventis Pasteur, Doylestown, PA), and HCMV Towne*var*RIT (passage 134, from Dr. Plotkin via Dr. Ed Mocarski, Stanford University), were propagated in human fibroblasts (HF) cultured in Dulbecco's modified Eagle's medium supplemented with 10% fetal calf serum (JRH Biosciences, Lenexa, Kans.). Recombinant HCMV, RC2.7EGFP, expressing enhanced green fluorescent protein (EGFP) (Clonetech, Palo Alto, CA), under the control of the major early 2.7 promoter, was constructed by cotransfection of plasmid pEAG2.7EGFP with a set of overlapping cosmid clones derived from HCMV Toledo (G.M. Duke, unpublished data).

### Plasmid constructions

**Figure 1:**
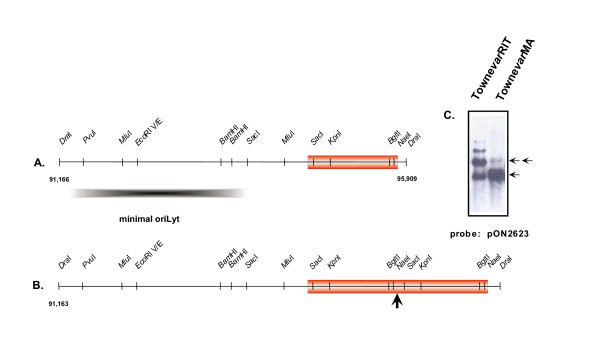
Schematic representation of amplified region near *ori*Lyt of the HCMV genome. **A**. Restriction enzyme map of minimal *ori*Lyt and adjacent region of heterogeneity (block). **B**. Region of heterogeneity shown as a dimer. Arrow indicates junction of the repeat segment. The number 91,166 to the left of the restriction map corresponds to the nucleotide position of the AD169 genome (EMBL accession number X17403). **C**. Autoradiograph of Southern blot utilizing a minimal *ori*Lyt probe, pON2623 (Kemble et al., 1996). Monomers and dimers are depicted with one and two arrows, respectively. Trimers and tetramers can be seen in the Towne*var*RIT viral stock.

Plasmid pON205 (Spaete and Mocarski, 1985), contains the Towne strain *a *sequence, was obtained from Ed Mocarski (Stanford University). pEAG2.7EGFP was derived by cloning the EGFP gene from plasmid pEGFP-N2 (Clonetech, Palo Alto, CA) between the *Eag*I and *Sma*I site of the β2.7 gene taken from Toledo (G.M. Duke, unpublished data). HCMV amplicon plasmid Tn9-8 was derived by inserting the 6 kpb *Dra*I fragment of Towne*var*RIT (corresponding to nucleotides 91,166 – 95,909 relative to AD169) (Figure 1B), spanning the HCMV oriLyt into the *Eco*RI site of pON205. Tn9-8 was partially sequenced by single-cycle and multicycle dideoxy-nucleotide chain termination method of Sanger *et al*., [51]. The plasmids designated Tn9-8GF5 and Tn9-8GF7 incorporating the EGFP gene with the HCMV Major Immediate Early (MIE) promoter and SV40 poly A sequence was isolated as a 2,334 bp *Nsi*I fragment from plasmid pEGFP-N2 (Clonetech, Palo Alto, CA), and cloned into the HCMV amplicon Tn9-8 at the *Pst*I site in both orientations. The *gpt *gene in Tn9-8-*gpt *was derived by cloning a PCR fragment from *Escherichia coli *DH5α using the primer pairs 5'CTGCAGCTAGTCTAGACTGGGACACTTCACATGAGC3'and 5'CTGCAGCTATGTATCTAGAGCCAGGCGTTGAAAAGATTA3'.

### Generation of viral stocks containing amplicons

Plasmid DNA was transfected by CaPO_4 _precipitation of approximately 4 μg of Tn9-8 amplicon DNA. The Tn9-8 DNA was transfected into approximately 1 × 10^6 ^passage 16 human fibroblast (HF) cells. At 24 hours post transfection, the cells were infected with CMV Towne at a multiplicity of infection (MOI) of 5 plaque forming units (PFU) per cell. Fresh medium was added to cells four days after infection and cells were harvested at 6 to 7 days post infection as described previously (Spaete and Frenkel, 1982). Virus stocks are prepared by three freeze-thaw cycles. Serial passages of amplicon-containing viral stocks on fresh HF cells were superinfected with CMV Towne as a helper virus at a MOI of 1.

### Southern blot analysis

Viral DNAs were digested with restriction enzyme, electrophoresed in 0.8% agarose gels, transferred to Hybond-N+ nylon membranes (Amersham Corp.), (Maniatis et al., 1989), and immobilized with a UV Crosslinker 1000 (Hoefer Scientific Instruments, San Francisco, CA). DNA on the membrane was probed with fluorescein-labeled pUC9 DNA using conditions previously described (Spaete and Mocarski, 1985).

### Isolation of CD34^+ ^cells and infection with CMV

The isolation of cord blood CD34+ stem cells was carried out by All Cells Inc. (Berkeley, CA) using CD34 Progenitor Cell Isolation Kit (Miltenyi Biotech, Auburn, CA). The positive selection of the CD34+ cells was carried out using hapten-conjugated antibody to CD34+ followed by anti-hapten antibody coupled to MACS Microbeads. The magnetically labeled cells are enriched on positive selection columns in the magnetic field. The purity of the CD34+ population was >95% as analyzed by flow cytometry. The purified CD34+ cells were suspended in Iscove's modified Dulbecco's Minimal Essential Medium containing 5% fetal bovine serum. 2 × 10^5 ^CD34+ cells were used for each infection with TN9-8GF5 amplicon containing stocks, RC2.7EGFP virus, CMV Towne virus, or uninfected cell control. The cells mixed with virus were centrifuged at 500 × g for 10 mins at room temperature and were then placed in 37°C water bath for one hour. Following this the cells were cultured in 6-well cell culture plates (Costar) for 18–72 hours. At the end of the incubation the cells were harvested for CD34 staining.

### EGFP expression and immunostaining for flow cytometric analysis

Amplicon containing viral stocks prepared from passage 1 were used to infect HF or human CD34+ cells maintained in 12 well culture plates. At 24 hour intervals post infection, the wells were observed for EGFP expression with a Nikon TE2000 microscope under UV illumination. Immunostaining for CD34+ cells was done using Phycoerythrin (PE)-conjugated anti-CD34 antibody (Becton Dickinson, San Jose, CA). Infected or control cells were incubated with 20 μl of PE-labeled anti-CD34 antibody for 45 minutes at room temperature and subsequently were washed twice with PBS containing 0.1% BSA. The cells were directly analyzed for EGFP and CD34+ staining on a FACSCalibur instrument (Becton Dickinson, San Jose, CA), at 18 and 36 hours post infection.

## Results

In order to exploit the natural tropism of HCMV for cells of the hematopoietic lineage, in a nonlytic manner, an HCMV amplicon i.e. a plasmid containing the HCMV oriLyt and *a *sequences was constructed. Theoretically, due to the large size of the HCMV genome, an amplicon derived from this virus should be able to carry the large DNA inserts and be capable of efficient introduction into hematopoietic cells by infection.

### Heterogeneity at oriLyt

During analysis of cosmid clones of HCMV strain Towne, sequence heterogeneity was observed in the *Eco*RI E fragment of Towne that was not present in the Toledo strain [27]. The *Eco*RI E region spans in part the complex oriLyt region [1,2,24,37]. Sequences in a 1.2 kbp repeat fragment were shown to give rise to the heterogeneity observed at this locus in the Towne genome (Figure 1). The coordinates of a single repeat unit starts at nucleotide 94,561 relative to the AD169 sequence and end at nucleotide 95,807 [10]. This segment can repeat at least three times in Towne strains from different passage histories (Fig. 1). This heterogeneity is different from the 189 bp repeat region previously described for the Towne strain oriLyt which occurs near the *Bam*HI sites in Figure 1 (nt 93337–93525 relative to AD169), [11,12]. Since Towne replicates to relatively high titers in cell culture, it was deemed advantageous to incorporate this heterogeneity in the origin containing sequences to be used in the amplicon construct.

### Construction of the HCMV amplicon

**Figure 2:**
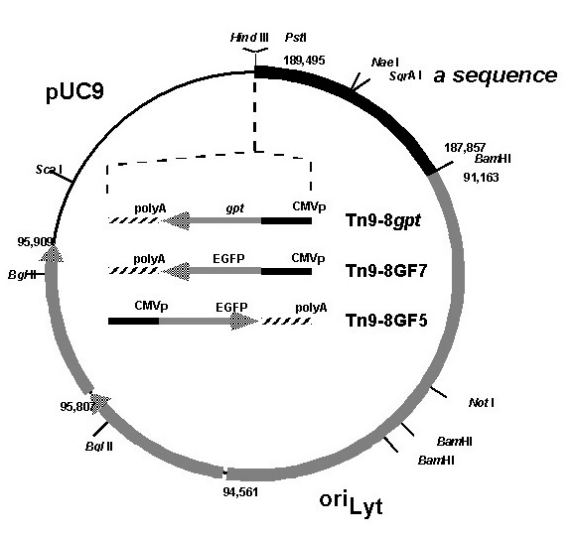
Schematic representation of the HCMV amplicon plasmids Tn9-8*gpt*, Tn9-8GF7 and Tn9-8GF5. The EGFP expression cassette was cloned in two orientations in the unique *Pst*I site in Tn9-8. The *gpt *expression cassette was cloned between the unique *Pst*I and *Hind*III sites.

As a test of the feasibility of the system, an HCMV amplicon was constructed which incorporated the two *cis*-acting functions required for the propagation of the defective virus genomes in the presence of helper virus (Figure 2). HCMV amplicon plasmid Tn9-8 was derived by inserting the 6 kpb *Dra*I fragment of Towne (corresponding to nucleotides 91,166–95,909 relative to AD169) [10] (Figure 1B), spanning the HCMV oriLyt into the *Eco*RI site of pON205. The resulting amplicon was designated Tn9-8 (Figure 2), and was partially sequenced to verify its structure.

### Generation of viral stocks containing amplicons

**Figure 3:**
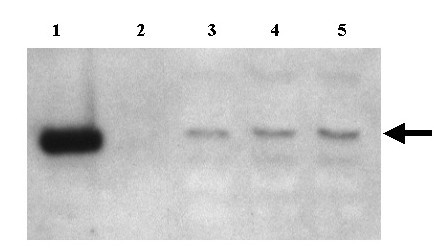
Southern blot analysis of passage 1 of HCMV amplicon DNA probed with plasmid pUC9. Lane 1. Plasmid Tn9-8 linearized with *Hind *III serves as a marker for correct migration of monomeric repeats. Lane 2. Plasmid Tn9-8 restricted with *Hind *III and *Dpn *I as a control for non-replicating DNA. Lanes 3–5. Infected cell DNAs restricted with *Hind *III and *Dpn *I. The signal in lanes 3–5 (arrow) demonstrates authentic replication and packaging of amplicon Tn9-8 in eucaryotic cells.

**Figure 4:**
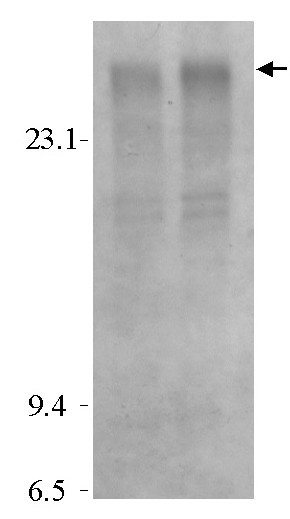
Southern blot analysis of high molecular weight HCMV amplicon DNA at passage 1 probed with plasmid pUC9. DNA prepared from passage 1 infected cells was digested with *Cla *I, *Xba *I, *Afl *II and *Dpn *I, Southern blotted and probed with plasmid pUC9. The samples are from those shown in Fig. 3, lanes 3 and 5. The high molecular weight DNA containing plasmid sequences (arrow) demonstrates the major hybridizing species migrating slower than the 23 kbp lambda DNA *Hind *III digest indicated as the marker on the left of the autoradiograph.

As a test of the ability of this construct to function as an amplicon, plasmid Tn9-8 was transfected in human fibroblast (HF) cells, and subsequently infected with HCMV Towne strain at an MOI of 5 to provide helper virus replication functions. Seven days later, infected cells were harvested, sonicated, and viral stocks were prepared for passage to fresh HF cells. Fresh HF cells were infected with the progeny of the transfection/infection and incubated for 7 days. The DNA from these infected cells was harvested (designated passage 1), restricted with *Hind*III and *Dpn*I, and Southern blotted [36]. Southern blot analyses of DNA demonstrated that Tn9-8 was susceptible to digestion with *Dpn*I, consistent with replication of the plasmid in bacteria (Figure 3, lane 2). In contrast, Tn9-8 in infected cells was resistant to *Dpn*I demonstrating that it had replicated in eucaryotic cells (Figure 3, lanes 3–5). This observation is consistent with replication and packaging of Tn9-8 into infectious virions. These results demonstrate that foreign DNA sequences, exemplified by the plasmid pUC9, can be introduced into defective genomes that are packaged and propagated in serially passaged virus stocks. To examine whether these results were the consequence of amplicon replication and packaging or integration of the amplicon plasmid into the helper virus, DNA prepared from passage 1 infected cells was digested with *Cla *I, *Xba *I, *Afl *II and *Dpn *I. These enzymes digested the Towne helper virus DNA to fragments no larger than 13.6 kbp but do not cut within Tn9-8. The amplicon DNA was significantly larger than 23 kbp consistent with the amplicon being replicated as a concatamer (Figure 4). This result indicated that the high molecular weight DNA containing plasmid sequences was packaged independently and was not integrated into helper virus.

Packaged defective viral genomes derived from Tn9-8 or a derivative containing a selectable marker (Tn9-8-*gpt*), were serially passaged in HF cells. Defective viruses could be detected at passage 3 when probed with plasmid pUC9; however, the copy number appeared to diminish upon serial passage (not shown). Selection with mycophenolic acid on Tn9-8-*gpt *amplicons did not enhance recovery.

### Rescue of monomeric repeat units in bacteria

**Figure 5:**
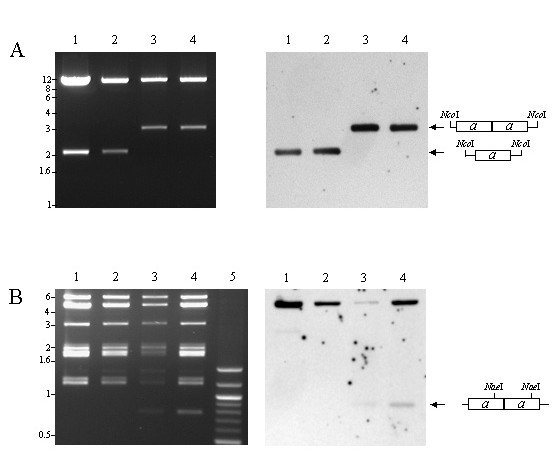
Tn9-8-*gpt *was rescued from concatamers following serial passage in HF cells. (A) Tn9-8-*gpt *was passaged in HF cells and monomer units were recovered by linearizing concatemeric DNA from serial passage 3 with *Hind*III and cloning in bacteria (lanes 2 and 4) and also by linearizing passage 2 DNA with *Pst*I and cloning in bacteria (lane 3). Lane 1 represents the unpassaged clone for comparison. Following rescue in bacteria, DNA was prepared and cut with *Nco*I. Fragments were separated on an agarose gel and visualized with ethidium bromide staining (left panel). The gel was transferred to a nylon membrane and probed with an *a *sequence specific probe (right panel). (B) Fragments from an *Nae*I digest were also separated on an agarose gel and visualized as in panel A. The gel was transferred to a nylon membrane and probed with an *a *sequence specific probe. Lane 5 shows a 100 bp ladder. Lanes 3 and 4 show a *ca*. 800 bp fragment that hybridizes to *a *sequences.

Concatemeric DNA was prepared from passage 2 and 3 virus stocks containing the defective virus genomes (Tn9-8-*gpt*), digested with *Pst *I and *Hind *III, respectively, in order to analyze monomeric repeat units. The *Hind *III-digested DNA was circularized by ligation and used to transform *E. coli *bacteria to analyze structure and to demonstrate shuttle vector capability between eucaryotic and bacterial hosts. A number of plasmids prepared from the rescue attempt had a restriction enzyme pattern indistinguishable from the input (Fig. 5A, lanes 1 and 2). Other plasmids however exhibited the expected restriction pattern consistent with a head-to-tail amplification of the *a *sequence (lanes 3 and 4). Digestion with *Nae*I produced a fragment of the predicted size of a unit length *a *sequence (762 bp), and this product hybridized with an *a *sequence specific probe (*Pst*I-*Sgr*AI fragment from Tn9-8), (Figs. 2 and 5B, lanes 3 and 4). This type of amplification has been readily seen in restriction enzyme digested DNA preparations of parental genomes of both HSV and HCMV [31,40,41,58,64,69] and has also been observed in HSV amplicons using Southern blot hybridizations [15].

### Expression of heterologous genes in an HCMV amplicon

**Figure 6:**
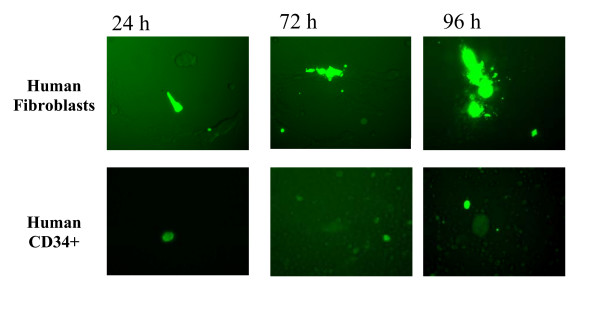
Fluorescent Microscopic Analysis of TN9-8GF5 amplicon infected cells. Human fibroblast cells (HF) or human cord blood CD34^+ ^cells were infected with TN9-8GF5 amplicon-containing stocks, or mock infected. Cells were observed at different time-points 24, 72 and 96 hrs post infection with TN9-8GF5 amplicon under the fluorescent microscope (Nikon TE2000 microscope). EGFP expressing fluorescent cells were observed in the TN9-8GF5 amplicon infected human fibroblast cells or human CD34+ cells at different time-points. Control uninfected cells were negative (not shown).

To demonstrate that the HCMV amplicon could be used as a vector system to support the expression of a foreign gene, EGFP under the transcriptional control of the HCMV major immediate early (MIE) promoter was used as test reporter gene. Two resulting amplicon plasmids designated Tn9-8GF5 and Tn9-8GF7 both expressed EGFP following transfection of HF cells in the absence of helper virus, as expected (not shown). Packaged amplicons were generated by introduction of Tn9-8GF5 into cells and infecting with HCMV 24 hours later at an MOI of 5. Transfection-derived viral stocks were passaged onto fresh HF cells supplemented with Towne helper virus at an MOI of 1. Viral stocks prepared from passage 1 were used to infect HF cells and grown on 12-well tissue culture plates. A limited number (ca. 0. 1%) of brightly fluorescing cells could be seen by microscopic examination at 24, 72 and 96 hours post-infection (Figure 6). This demonstrates that a foreign gene can be expressed in the context of a HCMV amplicon viral stock in infected HF cells.

To test the utility of the HCMV amplicon in gene therapy or gene delivery, we used packaged amplicons in viral stocks to infect and deliver an expressed gene into human CD34+ progenitor cells. Viral stocks containing amplicons carrying EGFP under the transcriptional control of the HCMV major immediate early (MIE) promoter prepared from passage 0 and passage 1 were used to infect CD34+ cells derived from cord blood. Starting at 24 h after infection, CD34+ cells were examined for EGFP expression by fluorescent microscopy. EGFP expression was observed in TN9-8GF5 amplicon-infected CD34+ cells starting at 24 h post-infection. The cells remained positive for EGFP expression for more than 96 hrs, at which point the cells were terminated (Figure 6).

**Figure 7:**
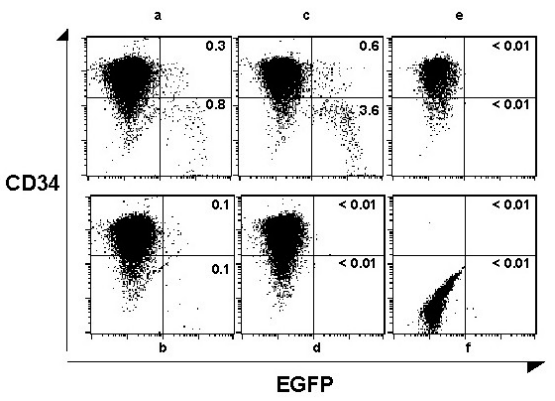
Flow cytometry analysis of human cord blood CD34^+ ^cells infected with CMV amplicon containing stocks, virus, or uninfected cell control. TN9-8GF5 amplicon (*a,b*), CMV-EGFP (RC2.7EGFP) virus (*c*), CMV (Towne) infected (*d*), or control uninfected human cord blood CD34 cells (*e,f*), were stained 36 hours post-infection with PE-antiCD34 antibody (*a-e*), or were left unstained (*f*), and were analyzed for two-color cytometry analysis using a FACS Calibur instrument. The dot-plots are generated using Cell Quest software and reveal the EGFP^+ ^cells populations. Numbers in the upper right and lower right quadrants indicate percentage of the EGFP^+^CD34^+ ^and EGFP^+^CD34^- ^cells respectively. A frequency lower than 0.01% is considered negative.

In a separate experiment, at 36 hours post infection, the cells were stained with PE-labeled anti-CD34 and analyzed for the CD34 marker and EGFP expression. EGFP expression was observed in the CD34+ population in the TN9-8GF5 amplicon (0.3%, 0.1%) (Figure 7a, &7b) or the CMV-EGFP virus (0.6%) (Figure 7c) at 36 hours post infection. The CMV Towne control-virus infected cells or uninfected CD34+ cell control were negative for EGFP expression (Figure 7d, &7e). A small population (7–12%) of the cells lost expression of the CD34+ marker upon *in vitro *culture. EGFP expression was also observed in a CD34(-) population infected with either TN9-8GF5 amplicon containing viral stocks (0.8%, 0.1%) or with the CMV-EGFP virus, RC2.7EGFP (3.6%) (Figure 7a, 7b &7c). The CMV Towne infected cells or uninfected control cell also had a significant CD34 negative population but were negative for EGFP expression (Figure 7d, and 7e). These results clearly demonstrate that CD34+ cells can be infected with replication competent or incompetent CMV vectors expressing a foreign gene.

## Conclusions

We have shown that a replication-defective virus vector system that is derived from HCMV is capable of delivering and expressing foreign genes in infected primary cells including progenitor stem cells such as human CD34+ cells. Further improvement and optimization of the system offers the potential to deliver gene-based therapies to multipotent cells.

### Advantages for use of the HCMV amplicon

Foremost among the advantages of the vector system we have described is the potential ability to efficiently infect and deliver genetic information to hematopoietic stem cells (CD34+) and other dividing and non-dividing cell types which may support HCMV infection [34,38,39,55,68]. Genetic hematological disorders such as thalassemias and sickle-cell anemia and other hemaglobinopathies could therefore be targeted for therapy with this strategy. Another potential advantage for the system is that vector DNA could possibly be maintained as an episome with minimal concern for the potential consequences of random integration of vector DNA (i.e. activation of oncogenes or inactivation of tumor suppressor genes). In order to insure efficient segregation as an episome, the EBV latent replication origin, oriP, and the transactivator, EBNA-1, could be added as was previously shown for another hybrid herpesvirus vector [71]. However such a modification may not be necessary because HCMV genomes appear to be carried continuously in cells of hematopoietic origin in infected individuals. Yet another potential advantage as with other herpesviral vectors, is that the HCMV vector system should have the capacity for very large inserts.

### Infection of CD34+ cells with HCMV

The infectivity of CD34+ cells from seropositive and seronegative subjects with HCMV has been tested both *in vivo *and *in vitro *[53]. Furthermore, hematopoietic stem cells are also reported as a site for HCMV latency. Efficient transduction of human CD34+ cells with retroviral and non-viral vectors has been unsatisfactory due to the lack of maintenance of high levels of expression of the transgene following engraftment of the engineered cells [16]. The HCMV MIE promoter may not be the right promoter for optimal expression in a CD34+ cells, since it has been shown that in the context of a lentiviral-based gene transfer system this promoter appeared to function less efficiently due to a cell-type specific expression defect [16]. The approaches to improving the efficiency of gene transfer into human cells have focused on improving gene delivery vectors and optimizing *ex vivo *culture conditions, which preserve the developmental properties of the stem cells [14,22]. Umbilical cord blood is recognized as a rich source of hematopoietic CD34+ stem cells [33]. In our experiments we used cord blood derived CD34+ cells for infection with HCMV amplicon containing stocks or HCMV-EGFP virus. However, bone marrow derived CD34+ cells have also been shown to be infectable *in vitro *with HCMV [34]. Gentry & Smith [21], reported a progressive loss of primitive cell properties including a reduction of CD34 expression upon *in vitro *culture of cord blood derived CD34+ cells. In a separate study, cord blood derived CD34+ cells cultured with IL-3 *in vitro *showed a progressive decline of the CD34+ population and more differentiated cells originating in the CD34(-) population [9]. In our experiments with >95% pure cord blood derived CD34+ cell population, a loss of CD34 expression in a small percent population (9–12%) of stem cells upon *in vitro *culture has been observed. EGFP expression was also seen in the CD34(-) population (Fig 7a, 7b, &7c). It is possible that HCMV infection of CD34 cells could induce cell differentiation and loss of primitive properties including reduction of CD34 expression.

Further studies of HCMV infection in CD34 cells will help in defining whether CD34+ infected cells undergo cell differentiation by increased expression of other markers such as CD33, CD38, HLA-DR or cytokines. It is also relevant to note here that HCMV virus carries homolog sequences for HLA-related and cytokine-related molecules and infection can induce cellular cytokines [5,8,19,32,44,46,50,66]. The HCMV amplicons contain only the *cis*-acting ori and packaging sequences, and have no structural gene sequences. However, amplicon containing viral stocks are a mixture with HCMV replication competent helper virus. HCMV induced cell-differentiating effect, if any, might be minimized using a helper virus-free amplicon system. In this regard, it should be possible to test a number of strategies to prepare helper virus-free stocks [17,63]. These preparations would be useful for therapeutic applications in immuno-compromised patients.

## Competing Interests

The authors of this publication are supported financially by salary and shares of MedImmune Inc., during the completion of this work. A patent application has been filed with the United States Patent Office relating to the content of this manuscript. The authors have assigned all rights of ownership to MedImmune Inc. The authors declare that they have no other competing interest.

## Authors' Contributions

KM carried out the expression analysis in human fibroblasts and CD34 cells. MNP & GMD generated amplicon stocks and MNP did the '*a*' sequence analysis. GWK provided critical intellectual input. RRS provided the original idea and experimental design as well as cloning of the amplicon, generation of amplicon stocks and Southern analysis.
